# Institutional Notes and News

**Published:** 1910-03-05

**Authors:** 


### Institutional Notes and News.

**Cancer Hospital, Fulham Road: Annual Report.**

The governors of the Cancer (Free) Hospital, Fulham Road, held their annual meeting last week, with Lord Northbrook, president of the hospital, in the chair. The pathological and research departments of the hospital are to be enlarged and known for the future as the Cancer Hospital Research Institute, the cost of which is reckoned at £7,000 for the building, and from one to two thousand for equipment. By the time this notice appears the work on this new department will probably have been begun. Mr. C. Ryall, in replying for the medical staff to the vote of thanks which had been passed for their work, had several interesting things to say about cancer and their efforts for its cure. He said that no period of life was immune from its attacks, and that no remedy was rejected by the staff in Fulham Road till it had been fairly and impartially tried. Only last year £200 had been spent on radium in order to test the value of radium treatment. His own belief was that surgery rather than medicine was the best weapon of science at the present time, but he pointed out that there were many cases, as a visit to the wards would show, where surgery could not be resorted to. Cases in the hospital could be divided into two classes, those likely to be amenable to surgical treatment, and those not so amenable which might be called chronic. The latter tended to fill up the wards, and he thought if the public could be taught to realise a few facts about the disease, and the horror it inflicted on those who could not secure hospital treatment, money would be raised by which new wards for chronic cases could be built, and many cases admitted which were now crying for treatment, but which the hospital's present accommodation was too limited to admit to the wards. Such wards might form a home in the country, and be specially set apart for the palliative treatment of chronic cases. In-patients for the year numbered 744, attendances in the out-patient department 12,567, and the daily average of occupied beds was 86. The total expenditure was £15,937, and the income excluding legacies, £9,161, leaving a deficit on the year's work of £6,775.

**Wimborne Cottage Hospital.**

Since 1904, as the latest annual report shows, the number of patients at the Wimborne Cottage Hospital has steadily increased. Last year 126 patients were admitted, and the average number of beds occupied was 8.5. It is, further, extremely creditable to know that after £80 had been placed to the reserve fund, there still remains a credit balance of 8s. 7d., all the more so because in 1908 an adverse balance had to be reported. The total receipts were £545, the expenditure £463, and the reserve fund now amounts to £223, and the invested funds are now £760. The new men's ward is to be christened the "Banks Ward," to commemorate the generosity of the late Miss Banks, who, besides contributing donations, left the hospital a legacy of £1,000 in her will.

**New Policy at Belfast Hospital for Sick Children.**

There is a point of interest to be found in the report of the last year's work at the Belfast Hospital for Sick Children, namely that a sum that runs into three figures has been saved in the last twelve months. The new policy, for it is only a year old, of having drugs and surgical dressings purchased and dispensed by the hospital's own dispenser, has proved a great success, for even after the dispenser's salary had been paid, the handsome sum of £115 remained, which was a clear saving to the institution. During the year 648 ward cases were received, and 11,753 attendances were made at the out-patient department. The daily average number of beds occupied was 35.84. Another noteworthy change was brought about with the approval of the Commissioners of Charitable Donations and Bequests by the sale of the old Convalescent Home, which realised £415, and this sum has been used for the purchase and decoration of a new house in Carrickfergus. When funds allow the Home to be in full working order, twenty children will be provided for without difficulty. The new Convalescent Home also will serve a useful purpose in removing chronic cases from the wards, which they now tend to occupy, and thereby will give to them a far better chance of recovery than they can have by remaining in the town. Both Mr. Cole, the new dispenser, and Miss Lockwood, the matron, were complimented in the report on their share in the year's work.

**Derbyshire Hospital for Women.**

The financial statement in the annual report of the Derbyshire Hospital for Women shows receipts £999, expenditure £869, with a balance on the right side of £130. This balance has resulted, it would seem, from the payments of the in-patients; admission to the hospital may be gained in two ways, either by a letter of recommendation or by the payment of a guinea a week. As the second of these methods had not been widely understood, the Committee recommended the alteration of Rule I. for the admission of in-patients. As amended, this reads: "Each in-patient who enters the hospital *without* a recommendation is required to pay, in advance, the sum of £1 1s. per week. Each in-patient who enters the hospital *with* a recommendation is required to pay, in advance, the sum of 1s. per day. A subscription of £2 2s. per annum entitles the subscriber to recommend one in-patient for a period not exceeding three weeks. The above sums provide not only for board and lodging, but also for the general expenses of the hospital, including medical attendance, medicine, nursing, coal, light, rates, taxes, etc. For a *private* ward an extra fee is charged. This extra fee is charged for the privacy only, the diet and nursing being precisely the same as in the general wards." Last year there were 146 in-patients and 1,945 attendances at the out-patient department. In recognition of the services of the matron, Miss Thomas, to the hospital the Committee had decided to raise her salary from Midsummer, 1909.

**Central London Ophthalmic.**

The Education Committee of the L.C.C. has sent a number of children with defective eyesight to the Central London Ophthalmic Hospital for treatment, and this explains the record figures, which are quoted in the 66th annual report, for the patients in all departments. The numbers were, in-patients 452, out-patients 13,718, and out-patient attendances 31,650. The delay which a variety of circumstances has caused in the rebuilding scheme is now over, and the work will, the report states, begin immediately. Two-thirds of the estimated cost, that is to say, £10,000, has been raised already. But these straightforward facts were not allowed to obscure the most interesting feature of the year, the influx of children sent for treatment already mentioned. Mr. T. Butlin Archer, indeed, took the opportunity afforded by his reply for the medical staff to the vote of thanks accorded to it, to criticise the L.C.C. He said that another institution had received a grant to establish a school clinic for the children, but that when the Central London offered to do its work on similar terms, the only reply received was that there were not enough children in the neighbourhood to justify the offer. This, in the face of the influx of children recorded was, he said, nothing but evasion, and some explanation was due to the hospital for the attitude the L.C.C. had taken up.

**Moorfields Eye Hospital, City Road.**

The annual reports of eye hospitals, especially the reports of those in the metropolis, have particular interest at the present time, in view of the fact that eye hospitals more than any other class have been affected by the medical inspection of school children. Mr. H. P. Sturgis, chairman of the Royal London Ophthalmic Hospital, stated at the annual governor's meeting the other day that arrangements had been made with the London County Council by which 12,000 children could now obtain treatment every year. Out-patients for the past year numbered on an average 380 a day, making a grand total of 116,178 attendances by 49,107 patients for the twelve months preceding. The average daily number of beds occupied was 100, and the in-patients had beaten all previous records by totalling 2,391. The hospital's income was £11,879, or in other words has been declining in the last year or two relatively to the expenditure, and the report was now compelled to chronicle a total deficit of £2,494. The good national work which the hospital is starting for London's children should make this, we hope, a merely temporary loss.

**Royal Westminster Ophthalmic Hospital.**

The Earl of Arran, as chairman of the Governing Body of the Royal Westminster Ophthalmic Hospital, in proposing the adoption of the report at the recent meeting, said that since 1906 the hospital had received no grant from the King's Fund. He attacked the ground—that they were not in debt, and so did not want the money—on which the Fund had thus acted by saying that the policy pursued by the hospital had always been one of avoiding expenditure unless the money was in hand to meet it, adding the sarcastic comment that this cause appeared to be in direct opposition to principles encouraged by King Edward's Hospital Fund. The Fund is thus placed in the Gilbertian situation of encouraging debt in the eyes of its critics whatever policy it may adopt. Certainly there is more plausibility in the charge from the opposite camp that to give to a hospital in debt is likely to make debt a comfortable and chronic experience. Still Lord Arran will not be taken too seriously. He congratulated the hospital on the continued prosperity of the School of Ophthalmic Surgery, which 53 students joined last year, of whom many were medical men in the services. The annual subscriptions had fallen off by £30, and the numbers of patients in both departments had increased. The report was adopted.

**King's College Hospital.**

The numbers of patients in the out- and in-patient departments of King's College Hospital for last year, as recorded in the annual report presented at the annual meeting of governors, are a little curious. The in-patients have gone up by 23, and the out-patients have gone down by 510. We imagine that a decrease in the numbers received at the out-patient department of a London hospital is not a very common experience. The in-patients anyway numbered 2,700. As in more than one instance, the ordinary income at this hospital showed a decline on previous years, the total being £13,774—a drop of nearly £2,500—and the legacies received were at the low figure of £513. At the end of the year, therefore, £6,400 was owing to the bank. The scheme for the Denmark Hill section of the hospital is advancing, £230,000 having been received towards the expenses of the building there. The Rev. Dr. Headlam, who presided, remarked, however, that £70,000 more is necessary to complete it. Within three years he hopes the work will be finished.

**Queen Charlotte's Lying-in Hospital.**

Queen Charlotte's Hospital has to complain like other institutions of a falling off in legacies, and this is the principal cause of the debt of £3,287, which has accumulated during the past three years. The report, presented at the governors' annual meeting, stated that 1,793 women were treated in the hospital last year, and 2,333 others were attended and nursed in their own homes. In the light of the above it is not surprising that the accounts show a deficiency on the year of £1,570. The actual figures are £5,580 credited to income, and £7,151 debited to expenditure. We trust that the appeal of Sir Samuel Scott, M.P., who presided, for further support, will be backed up and answered; certainly the two classes of hospitals which excite, perhaps, more sympathy in the public mind than any other are those which nurse children and those which attend to women in childbirth. It should not be difficult to show to-day, when the newspapers are full of the rising generations, what small likelihood they have of surviving if the hospitals cannot take in the mothers at that time.

**Italian Hospital, Queen Square.**

The patients received by the Italian Hospital, Queen Square, during the year are shown by the annual report, which was presented at the governors' meeting the other day, to bear a large proportion of British subjects. So, should a narrow patriotism ever have prevented any members of the giving public from extending their charity to this institution, they had better realise how many subjects of the King are treated there. The in-patients numbered 877 and the out-patients 4,401, making a grand total of all classes of 5,278. The accounts showed an income of £2,722, of which £434 was the result of annual subscription and £100 the customary annual grant from the Italian Government. The total expenditure, which included sums spent on necessary structural repairs, was £3,302, or, in other words, the income had fallen short by £580. It should be remembered that the income has fallen off last year because of a special loss: the unavoidable postponement of the entertainment given each year as a rule by the Italian Chamber of Commerce.

